# Harrodsburg Springs

**Published:** 1840

**Authors:** 


### HARRODSBURG SPRINGS.

We understand that Dr. Graham, the enterprizing and obliging proprietor of this popular watering place, has lately made large additions to his previous means of accommodation. In many respects, this is the most elegible place of summer resort in the valley of the Mississippi—of the whole, it is, indeed, the most highly improved by art. For the information of such of our readers as have not seen an account of the composition of the waters, we may state that the active ingredient, is *sulphate of magnesia.* D.

